# Association between Natural Resources for Outdoor Activities and Physical Inactivity: Results from the Contiguous United States

**DOI:** 10.3390/ijerph13080830

**Published:** 2016-08-17

**Authors:** Yan Jiang, Yongping Yuan, Anne Neale, Laura Jackson, Megan Mehaffey

**Affiliations:** USEPA Office of Research and Development, National Exposure Research Laboratory, 109 T. W. Alexander Dr., Research Triangle Park, NC 27711, USA; enaj1125@gmail.com (Y.J.); neale.anne@epa.gov (A.N.); jackson.laura@epa.gov (L.J.); mehaffey.megan@epa.gov (M.M.)

**Keywords:** protected areas, community health, physical inactivity, spatial autocorrelation, spatial lag model

## Abstract

Protected areas including national/state parks and recreational waters are excellent natural resources that promote physical activity and interaction with Nature, which can relieve stress and reduce disease risk. Despite their importance, however, their contribution to human health has not been properly quantified. This paper seeks to evaluate quantitatively how national/state parks and recreational waters are associated with human health and well-being, taking into account of the spatial dependence of environmental variables for the contiguous U.S., at the county level. First, we describe available natural resources for outdoor activities (ANROA), using national databases that include features from the Protected Areas Database, NAVSTREETS, and ATTAINSGEO 305(b) Waters. We then use spatial regression techniques to explore the association of ANROA and socioeconomic status factors on physical inactivity rates. Finally, we use variance analysis to analyze ANROA’s influence on income-related health inequality. We found a significantly negative association between ANROA and the rate of physical inactivity: ANROA and the spatial effect explained 69%, nationwide, of the variation in physical inactivity. Physical inactivity rate showed a strong spatial dependence—influenced not only by its own in-county ANROA, but also by that of its neighbors ANROA. Furthermore, community groups at the same income level and with the highest ANROA, always had the lowest physical inactivity rate. This finding may help to guide future land use planning and community development that will benefit human health and well-being.

## 1. Introduction

Promoting physical activity has come to global attention due to death and disease caused by inactivity [1,2,3,4]. The Lancet Physical Inactivity Series reported one-third of adults and 80% of adolescents around the world do not meet recommended levels of daily physical activity [5]. The natural environment has thus received more and more attention as a way to promote activity. In the Netherlands, researchers studied the relationship between green space in people’s living environment and their perceived general health, and found the percentage of green space within one to three kilometers’ radius around their postal code coordinates had a significantly positive effect on their perceived general health [6]. A Canadian study found that youth who had access to a playground in a nearby park were five times more likely to be at a healthy weight than to be overweight or obese [7]. U.S. research also indicated that residential proximity to parks was a critical determinant of park use and leisure exercise [8].

Previous studies that quantified a link between the natural environment and physical activity and positive health outcomes focused mostly on local parks. To our knowledge, no study has examined the association between federal and state protected areas (e.g., national/state parks and recreational waters) and physical activity rate. The Pan American Health Organization/World Health Organization (WHO) and National Park Services have called for research to evaluate how national recreational areas improve human health and well-being. In addition, previous studies have often used conventional regression methods that assume observations are independent of one another [6,7,8,9]. National environmental observations are spatially dependent or continuous [10], however. For example, natural resources in one county could be used by its own residents as well as those from neighboring counties [11,12]. Ignoring the spatial dependency between observations produces estimates that are biased and inaccurate [11,13]; thus, using spatial regression, where natural environment is treated as spatially continuous, is needed for more comprehensive evaluation of the connectivity between the natural environment and well-being of humans.

Human health and well-being are also affected by socioeconomic factors such as income, race and age [14,15]. It is well-documented that adults whose family income is above the poverty level are more likely to meet the Physical Activity Guideline for aerobic activity than those whose income is near at or below the poverty level [16]. Another study argued that obesity and related health risks are greatest for low-income, Black, and Latino communities where natural resources for outdoor activities are least available [17]. Recently, researchers also examined how to decrease health inequality of low income groups by providing natural environment resources. A study in England found that populations with the greatest access to green space had the weakest associations between income-related deprivation and all-cause and circulatory disease mortality [18]. Although providing access to natural environments to address socioeconomic inequality in health has begun to receive attention, relevant research is limited.

The goal of our study was to investigate contributions of national/state parks and recreational waters to human health and well-being from a spatially-continuous perspective, focusing on physical inactivity. We also investigated whether the association between national/state parks and physical inactivity was similar across all income levels, to understand if the health inequality of low income groups can be reduced by increasing their access to natural resources for outdoor activities.

## 2. Materials and Methods 

### 2.1. Definition and Estimation of Available Natural Resources for Outdoor Activities

To better characterize national/state parks and recreational waters, we defined them as available natural resources for outdoor activities (ANROA). In such areas, people can be in contact with nature and have easy access to outdoor activities such as hiking, mountain climbing, scenery viewing, fishing, hunting, picnicking, biking, swimming, etc. To make the ANROA dataset as complete as possible, we selected recreational sites that are open to the public at no cost as well as those accessible through permit registration or fee. Three databases were used to estimate the ANROA: the Protected Areas Database of the United States (PAD-US) (version 1.3, publication date: 30 November 2012); NAVSTREETS; and ATTAINSGEO 305(b). The PAD-US is an inventory of marine and terrestrial protected areas dedicated to preserving biological diversity, and other natural, recreational and cultural uses including both “Open” (no special requirements for public access) and “Restricted” (a permit is required for access) (PAD-US 2012) [19]. NAVSTREETS contains recreational features such as national, state, and city parks, as well as water parks. Lastly, we used ATTAINSGEO 305(b) Assessed Waters by Assessed Uses to identify the spatial extent of recreational water features listed under Section 305(b) of the Clean Water Act [20]. Combining those three databases is unique and comprehensive. ANROA now is an expansion of national/state parks and recreational waters; examples are Yosemite National Park, Lake Tahoe, and Kaibab National Forest. The study area includes the contiguous United States (48 states plus Washington, D.C., on the continent of North America). When calculating the ANROA, overlapping features of the three databases were removed, using the ArcGIS Dissolve Tool, so no duplicate counting occurred [21]. All the spatial analysis in this study was conducted using the geographic information system—ESRI ArcGIS. To consider the influence of natural resources for outdoor activities not included in any county’s boundaries—particularly coastal resources—a dummy variable was used in modeling to examine their potential association. Counties with a coastline were selected based on the NOAA coastal line. For analysis, counties with a coastline had a dummy variable equal to 1, and those without had a dummy variable equal to 0. The ANROA was calculated using the total natural recreational area within a county’s borders, divided by total county area. ArcGIS Spatial Analyst determined total county area and the natural recreational area within it. Counties were ranked by ANROA value, after which they were divided into equal categories: lowest, middle and upper thirds.

### 2.2. Health and Socioeconomic Data

Physical activity defined by the WHO as any bodily movement produced by skeletal muscles that requires energy expenditure—including activities undertaken while working, playing, carrying out household chores, travelling, and engaging in recreational pursuits [22]. Physical inactivity is a term used to identify people who do not get the recommended level of regular physical activity. Age-adjusted, leisure-time physical inactivity prevalence data were obtained from the U.S. Centers for Disease Control and Prevention’s (CDC’s) Behavioral Risk Factor Surveillance System for 2008–2011 [23]. The CDC is a federal agency that provides U.S. public health statistics for diseases, pregnancies, births, aging, and mortality. Data published on its website are available for public use (http://www.cdc.gov/diabetes/atlas/countydata/County_ListofIndicators.html). Data for leisure-time physical inactivity were compiled from self-reports. County demographic data (e.g., household income) were obtained from the U.S. Census Bureau’s 2008–2012 ACS 5-Year Summary File (Table B19001). Counties were placed in a group based on percentage of households with low-income: Group A had fewer than 20% of households with low income; Group B had 20%–50% households with low income; and Group C had more than 50% of households with low income. The threshold for low income was $30,000/year which is two times the U.S. poverty level [24].

### 2.3. Statistical Analysis and Modeling

To model the physical inactivity rate as a function of ANROA and socioeconomic variables, we performed two analyses: an ordinary least-squares (OLS) regression and a spatial lag regression. We also analyzed the potential influence of ANROA on different income community groups, using analysis of variance (ANOVA).

We estimated the OLS regression model as y = Xβ + ε, where y is a vector of observations on the dependent variable (physical inactivity rate); X is a matrix of independent variable (ANROA); β is a vector of coefficients; and ε is a vector of random errors [25]. Despite the popularity of the OLS approach, problems with spatial autocorrelation limits its application in analyzing spatial relationships. Residents in one region often access natural resources for outdoor activities in their own region as well as neighboring regions, so it is important to consider spatial dependence inherent in the data. Furthermore, spatial autocorrelation can also lead to correlation of error terms which means assumptions of OLS regression would be violated and estimates derived from it are likely biased [13]. To assess spatial autocorrelation, we therefore calculated the Moran’s I score, using ArcGIS to examine clustering of the residuals from the OLS model. The Moran’s I values typically range from −1 (indicating perfect dispersion) to +1 (perfect clustering). To control for spatial autocorrelation, we used spatial regression models to examine relationships of selected variables. Assuming that values of y in one unit are directly influenced by values of y in its neighbor(s), we used a spatial lag model (Equation (1)) to inspect the relationship with GeoDa [26,27]:

(1) Y=Xβ+ρWy+ ϵ

where ***X*** is a matrix of observations on the independent variables; ***β*** is a vector of regression coefficients; ***W*** is the spatial weights matrix representing the geography of the observation units; ***ϵ*** is a vector of error terms (and should be identically independently distributed); and ***ρ*** is the spatial autoregressive coefficient. Although ***β*** represents only the direct effect of xi on yi, which is the direct effect of ANROA on the physical inactivity rate in the spatial lag model (Equation (1)), the spatial lagged term leads to a chain-reaction. Change in xi will therefore affect other counties and, in turn, affect yi through the impact of ***y***. The spatial lag equation could thus be rewritten as Equation (2), which accounts for both direct and indirect effects of change by moving all terms involving the dependent variable y to the left-hand side.

(2) $$E\left(y\right)={\left(1-\rho W\right)}^{-1}X\beta$$

The spatial lag regression model explores potential relationships between dependent variables of physical inactivity rate and the independent variable ANROA, taking into consideration the spatial effect (Spatial Lag Model I (SL I)). We also used percentage of low-income households in a county to represent socioeconomic status, and added this variable to the model to examine potential association (referred to as Spatial Lag Model II). The Spatial Lag Model II (SL II) therefore examined three potential county health influences: natural environment (ANROA), socioeconomics (percent low household income population), and spatial effect (spatial autocorrelation). Log-likelihood, Akaike information criterion (AIC), and Bayesian information criterion (BIC) [25] determined how well each model fit the data and explained the quality of the modeled relationship between ANROA and physical inactivity rate. Both ACI and BIC deal with trade-offs between goodness-of-fit and complexity of the model, and their values depend on the number of observations and parameters. The higher the Log-Likelihood and the lower the AIC and BIC values, the better the model’s quality in fitting data. We used the GeoDa tool and R for all regression analyses [26,28].

Finally, we explored the potential influence of ANROA on income-related health inequality using ANOVA tests. Two-way ANOVA examined whether there were significant differences in physical inactivity rates between counties with low, moderate and high household income levels and ANROA. One-way ANOVA further evaluated the association of ANROA on physical inactivity rate for each income group; the significance level for all tests was 0.05. Diagnostic plots examined the patterns and normality of residuals. Analyses were conducted using R and differences were considered significant if *p* ≤ 0.05. Grouping criteria are described in Health and Socioeconomic Data [24].

## 3. Results

**Figure 1:**
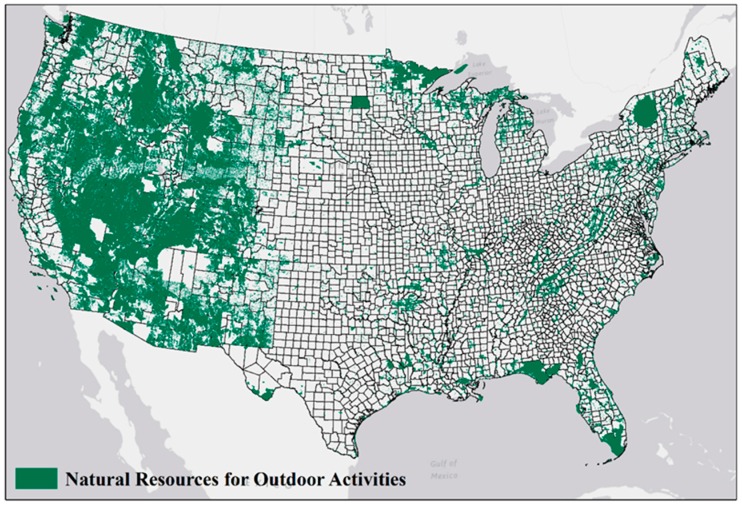
Geographic Distributions of Contiguous U.S. Natural Resources for Outdoor Activities.

ANROA was highest in the West, moderate in the Northeast and coastal areas, and lowest in the Midwest and South (Figure 1). 

**Figure 2:**
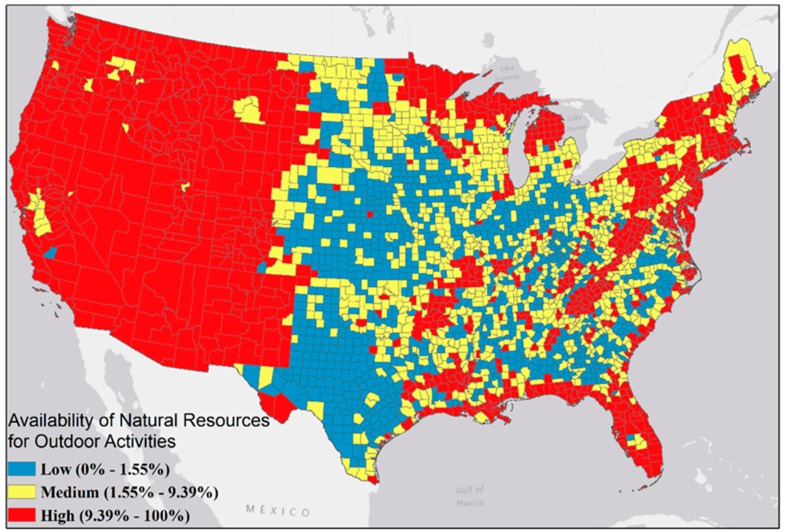
Classification of ANROA for the Contiguous U.S. Counties.

Across contiguous U.S. counties, ANROA ranged from 0% to 100%, with mean = 13.50%; median = 3.85%). One-third of U.S. counties had an ANROA value ≤ 1.55%, and one-third had an ANROA ≥ 9.39%. Counties were classified as low, medium, and high for ANROA, with medium ranging from 1.55% to 9.39% (Figure 2).

**Figure 3:**
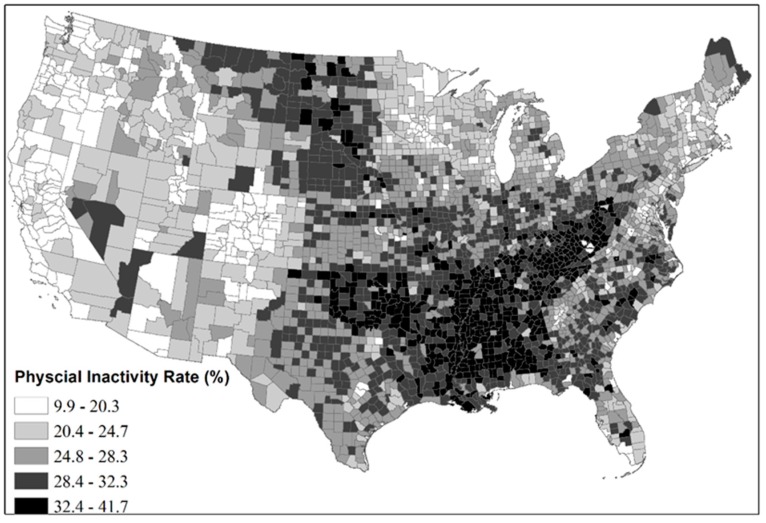
Geographic Distributions of Average Age-Adjusted Physical Inactivity Rate in Contiguous U.S. Counties (2008 to 2011; rates were grouped by natural breaks classification).

Average age-adjusted physical inactivity rate showed similar spatial patterns (Figure 3). The highest inactivity ranged from around 30% to 40% (Figure 3). People living in the West, Northeast, upper Midwest, and coastal areas appeared to be more active than those from other regions.

Counties with higher ANROA generally had a lower physical inactivity rate. For example, Colorado’s 64 counties had an average ANROA of 40.1% (High Group in Figure 2) and an average inactivity rate of 17.6% (max 24.5%), both of which are relatively low nationally (Figure 3). On the other hand, Southeastern states such as Alabama had a lower average ANROA (4.7%) and an average inactivity rate of 32.1% (max 36.5%), both of which are relatively high nationally (Figure 3). Besides these state differences, association between ANROA and physical inactivity within a state is also significant For instance, counties in central Florida have an average ANROA of 32.1% and an average physical inactivity rate of 28.6%; by comparison, coastal counties have a higher average ANROA of 38.9% and an average physical inactivity rate of 24.9%.

OLS model quantitatively examined the association between ANROA and physical inactivity rate, and presence of their spatial autocorrelation. Results showed that ANROA was a significant predictor (*p* values < 0.0001) of physical inactivity rate across all U.S. counties, accounting for 16% of the variation of physical inactivity (Table 1). Meanwhile, spatial autocorrelation tests indicated strong spatial dependence patterns with Moran’s I values of 0.65. The higher Moran’s I values are, the stronger are spatial clustering effects, with 1 indicating perfect clustering. High Moran’s I values in OLS models thus indicate strong spatial clustering effects associated with the geographic patterns of variables analyzed in this study.

SL I examined the association between ANROA and the physical inactivity rate while considering their spatial autocorrelation. Moran’s I values on residuals are approximate to zero for SL I, as shown in Table 1, indicating spatial autocorrelation was well-addressed. The R^2^ is much improved for all three physical inactivity rates which indicates increased precision of the spatial regression model. Increased Log-Likelihood and decreased AIC and BIC scores in the spatial lag models also indicated improved model performance (i.e., better representation of their associations) [29,30]. Although the absolute values of coefficients (β) decreased between the use of OLS and SL I for physical inactivity rate, β coefficients in the spatial lag models represent only the immediate effect of ANROA on physical inactivity rate. Total effect of the ANROA on physical inactivity rate includes both direct (Xβ) and indirect chain effects (spatial lagged term ρWy), as shown in Equation (1). The much improved R^2^ values from OLS (0.16 for physical inactivity rate) to SL I (0.69) imply that much stronger associations between ANROA and three physical inactivity rate exist, a fact which was not discovered by the OLS model. Building on SL I, the SL II examined the association of ANROA and household income level with physical inactivity rate by adding the percent of low-income households, and the model’s fitness was further improved, as shown in Table 1.

**Figure 4:**
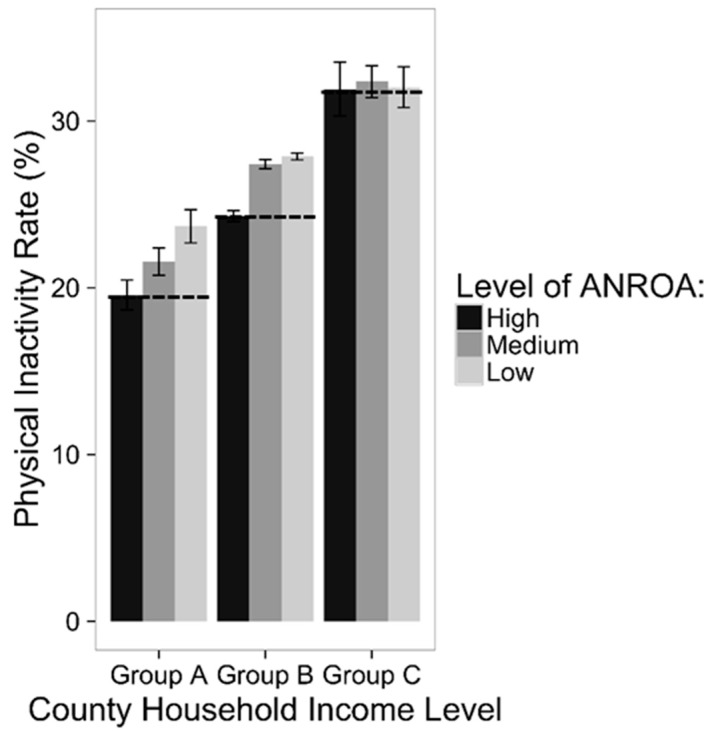
Average Age-adjusted Rate of Physical Inactivity among ANROA and Income Groups (Group A: low income pop < 20%; Group B: 20% < low income pop < 50%; Group C: low income pop > 50%. Error bar indicates 95% confidence interval; the classification of ANROA is consistent with Figure 2).

We explored potential association of ANROA and household income levels on physical inactivity using ANOVA tests. The two-way ANOVA test showed physical inactivity rate differs significantly (*p* value < 0.05) across groups of ANROA, as well as across groups of household income (Table 2). Counties with the highest ANROA have the lowest physical inactivity rate (Figure 4), although the difference is not statistically significant for Group C which has the highest percentage of low income households. The group with the lowest percentage of low income household (Group A) has the lowest physical inactivity rate. Meanwhile, we detected a significant interaction effect (*p* value < 0.05) between ANROA and household income in relation to physical inactivity, which means the association of ANROA with physical inactivity rate also depends on household income level. 

We further evaluated whether (and how) the association of ANROA on physical inactivity rate varied for different household income groups using the one-way ANOVA test. For income groups A and B, counties with the highest ANROA have the lowest physical inactivity rate, followed by counties with medium ANROA; counties with the lowest ANROA have the highest physical inactivity rate. Differences were significant (*p* value << 0.001). For income group C, the difference is not significant (*p* value = 0.85), although counties with the highest ANROA had the lowest physical inactivity rate. In addition, counties with medium levels of ANROA have a higher physical inactivity rate than counties with low ANROAs (Figure 4).

## 4. Discussion

This newly-developed data layer presenting distribution of available natural resources for outdoor activities in the contiguous U.S. is significant in several ways. First, the information can be used in studies assessing how natural resources being utilized for outdoor activities can benefit human health and well-being. Secondly, the information can guide land use planning and community development. Finally, the information may help to enhance local communities’ economies because jobs may be created for businesses related to outdoor activities.

Across the contiguous U.S, locations with higher ANROA appear to have lower physical inactivity rates. Statistical analysis and modeling demonstrate significantly negative associations as well as strong spatial dependency. Spatial dependency suggests that the health of a community is not only influenced by its in-county ANROA level, but also by that of its neighboring counties’ ANROAs and physical inactivity rates. The SL II, which analyzed the association of ANROA and household income level with community physical inactivity rate, further improved the fitness of the model (Table 1). It shows that physical inactivity rates are not only affected by ANROA, but also household income. Still, one may continue to question the impact that ANROA has on physical inactivity, given the low spatial autoregressive coefficient (ρ: −0.09, −0.02, −0.03) listed in Table 1. The explanation is that the spatial model assigns variation to the spatial lag of the dependent variable, producing smaller estimates, which has been reported frequently in other national-scale modeling studies [11,13,27,31]. We would like to clarify that we discovered a statistically significant association between the ANROA and physical inactivity in this study, not a direct cause-and-effect relationship. Given that human physical inactivity is influenced by a variety of factors, our study sought to detect a pattern and acknowledge the contribution to human health and well-being by natural resources for outdoor activities. In addition, physical inactivity varied significantly when stratified by income or ANROA levels, based on our ANOVA test. Physical inactivity rates differ significantly (*p* value < 0.05) across household income groups as well as across ANROA groups. Therefore, income-related inequality in health conditions might be reduced by providing low-income populations with more ANROA, such as issuing them annual free passes. The statistical significance between ANROA and physical inactivity rates warrants additional research.

Most previous research on health benefits of natural resources focused exclusively on green space or city parks [6,7,8,9], with few examining federal and state protected areas such as national/state parks and recreational waters. Our study fills this gap by examining the association between federal and state protected areas that provide excellent opportunities for vigorous outdoor activities and engaging with nature [32,33], and human health. We combined features from three national databases: Protected Areas Database, NAVSTREETS, and ATTAINSGEO 305(b) Waters. To make this study unique and thorough, we considered health benefits of federal and state protected areas, national/state parks and recreational waters, along with city parks. Our study provided not only qualitative descriptions of the relationship between ANROA and physical inactivity rate, as others have, but also quantitative evidence of how natural resources for outdoor activities can lead to a more active lifestyle [34,35,36]. Furthermore, we investigated spatial effects on physical inactivity rates, while previous studies have mostly used classic OLS regression or statistical tests which failed to examine spatial autocorrelation [6,7,8,9]. Ignoring spatial autocorrelation of variables led to inaccurate descriptions of their associations: for instance, people living close to a county’s boundary may engage in physical activities using the ANROA in their own county as well as the neighboring county. Ignoring such spatial autocorrelation means that use of the ANROA in a neighboring county is not considered.

Social and economic processes often interconnect with lifestyle, family and peer influences and can cluster in regions, meaning that inactivity rates in one county may affect those of neighboring counties due to social influences. Therefore, counties surrounded by counties with high ANROA and high income households are more likely to have low rates of physical inactivity. These findings are consistent with recent studies in England and The Netherlands that demonstrated reduction in health-related indicators for people with greater access to natural resources for outdoor activities [18,37].

Knowing the positive impact of natural resources for outdoor activities on community health can help land use planning and natural resources for outdoor activities protection. ANROA should be considered by community planners when endeavoring to improve community health, especially for lower socioeconomic groups. Policy-makers should strive to protect natural resources and limit their future development [38]. In addition, some natural resources for outdoor activities require fees to access which can be a barrier for low-income groups. Policy-makers should consider removing this barrier by eliminating such access fees.

Natural resources for outdoor activities are also vital for enhancing communities’ economies. Western non-metropolitan U.S. counties with significant amounts of federal protected lands added jobs more than four times faster than counties without protected lands [39]. In southeastern Pennsylvania, nearly 200,000 acres of protected open land generates economic benefits by helping to avoid nearly $800 million in direct and indirect medical costs, and supporting nearly 7000 jobs [40]. In 2011, economists and academics across the U.S. sent a letter to President Obama urging him to create jobs and support businesses by investing in public land infrastructure and establishing new protected areas such as parks, wilderness, and monuments [41].

Our study’s limitations are that, while we demonstrated linkages between ANROA and physical inactivity rate, we cannot exclude the possibility that active people choose to live where abundant recreational resources are available. We doubt, however, that all active people can choose freely where to live. Since it has been a challenge to derive a causal model for neighborhood effects [42], this paper focuses on association rather than cause-and-effect. It is impossible to control for direct selection on dependent variables in a cross-sectional design [6,43]. Although we combined three national databases to estimate the ANROA—which is as inclusive as possible—we might have overlooked smaller, local parks not included in those databases. With the exception of coastal recreational resources, ANROAs which did not fall in any county’s boundaries (e.g., international borders) were not included in this analysis. Our estimates of regression coefficients were also likely conservative due to limitations of physical inactivity rate data; for instance, the CDC indicates less than half (48%) of all adults meet the 2008 Physical Activity Guidelines in the United States, but self-reported physical inactivity rates for counties in our study were usually below even that. And since age adjustments were based on the 2000 Census, rates tended to be underestimated due to population distribution changes between 2000 and 2010 [44]. Finally, our analysis did not investigate other reported sociodemographic influences such as social factors (age, sex, health status, self-efficacy, and motivation) and physical environmental characteristics such as urban planning and transportation systems [15]. More research could examine these associations at finer spatial resolution (e.g., sub-county level) if such data sets become available in the future. Going beyond an ecological study, the context and scientific background support validity of the associations shown, but the study design limits the strength of the conclusions.

Despite these limitations, our study uses multiple national natural recreational datasets and complements findings from local-scale research. To our knowledge, this analysis is the first to quantify association of ANROA and socioeconomic status with physical inactivity rate, using spatial regression methods for the contiguous U.S. Our dataset is complete and our analysis is thorough. The spatial regression method illustrated connections between natural environments, socioeconomic, and public health processes among communities which had not been addressed in previous studies. We emphasize the importance of ANROA to human health and suggest that there is potential for promoting health within the low-income community by providing that population with greater access to natural resources for outdoor activities. Statistical significance between ANROA and physical inactivity rate warrants additional research into that relationship.

## 5. Conclusions

In a national analysis, this study revealed a strong negative association between ANROA and physical inactivity rates, respectively. In particular, communities with the highest ANROA have the lowest physical inactivity rates, regardless of income level. Additional research is warranted to better understand how ANROA may contribute to reduce health disparities between income levels.

## Figures and Tables

**Table 1:** OLS and Spatial Lag Models of Physical Inactivity Rate.

Model	Variable	Coefficients (β)	Pseudo-R^2^	Moran’s I Score	Log Likelihood	AIC *	BIC *
OLS	Constant	27.71	0.16	0.65	–9024.8	18,053.7	18,065.7
	ANROA	–0.09					
SL I **	Constant	5.98					
	ANROA	–0.02					
	ρ * of inactivity rate	0.79	0.69	–0.059	–7720.5	15,447.0	15,465.2
SL II **	Constant	4.54					
	ANROA	–0.03					
	%low-income household	0.14	0.73	–0.0017	–7433.2	14,874.4	14,898.6
	ρ * of inactivity rate	0.64					

* ρ is the spatial autoregressive coefficient; AIC is Akaike information criterion; and BIC is Bayesian information criterion; ** Spatial Lag Model I (SL I) examines influences of ANROA and spatial effect on physical inactivity rate; Spatial Lag Model II (SL II) examines influences of ANROA, spatial effect, and household income level on physical inactivity rate. All *p* values of coefficients (β) << 0.001.

**Table 2:** Two-way ANOVA Test of physical inactivity.

Factors	*p*-Values
Main effect: ANROA	<<0.001
Main effect: Household Income	<<0.001
Interaction Effect: ANROA & Household	0.0039
